# Influence of designated properties on the characteristics of dombeya buettneri fiber/graphite hybrid reinforced polypropylene composites

**DOI:** 10.1038/s41598-020-68033-y

**Published:** 2020-07-06

**Authors:** I. O. Oladele, M. O. Oladejo, A. A. Adediran, B. A. Makinde-Isola, A. F. Owa, E. T. Akinlabi

**Affiliations:** 1 Department of Metallurgical and Materials Engineering, Federal University of Technology, Akure, Ondo State Nigeria; 2 Department of Mechanical Engineering, Landmark University, Omu-Aran, PMB 1001 Kwara State Nigeria; 3 Department of Metallurgical and Materials Engineering, Federal University Oye-Ekiti, Ekiti State, Nigeria; 4 Department of Mechanical Engineering Science, University of Johannesburg, Johannesburg, South Africa

**Keywords:** Engineering, Materials science

## Abstract

This research presents the behavior of dombeya buettneri fiber/graphite hybrid composites which was studied to harness a favorable balance between the inherent advantages and disadvantages of natural and synthetic reinforcements. The fibers after extraction were chemically treated for surface modification. The composite was developed using compression molding process by randomly dispersing the reinforcements in the polypropylene matrix in predetermined proportions. The developed samples were tested to ascertain the response of the materials to the selected properties. Experimental results showed that hybrid composite sample C which is a blend of 12 wt% dombeya buettneri fiber (DBF) and 8 wt% graphite particle (GP) gave enhanced results in many of the properties which includes; hardness, impact, thermal insulation and abrasion resistance properties. Also, the hybrid composites sample denoted as sample E which is the blend of 6 wt% DBF and 14 wt% GP produce higher enhancement in the flexural properties and Young’s Modulus of Elasticity than other samples. Composite sample reinforced with dombeya buettneri fiber as single reinforced composites performed more in ultimate tensile strength compared to other samples while graphite particle reinforced sample emerges as the best in thermal conductivity. Diffusion of water into the composites also obeys Fick’s law where sample C was seen to be the best among the composites. It was therefore, discovered that the synergy between the two reinforcements has encouraged the improvement of polypropylene (PP) properties in a unique mode.

## Introduction

The choice of natural reinforcement materials in the design of polymeric based composites has gained significant attention from researchers^1,2^. The characteristics properties such as good dimensional stability, ease of design, corrosion resistance, good mechanical strength and lighter weight makes these set of materials desirable over the traditional materials^3^. They have been successfully utilized in the development of composite materials with good property when compared with synthetic fibers^4^. In composite formation, the physical and chemical identities of the fibers and matrix are retained, while the composite formed, possess a blend of properties not attainable by each constituents. It has been established that in fiber reinforced polymer composite, fibers are the main load-carrying members. Notable characteristics of the matrix material are; to transfer stresses between the fibers, serves as a barrier against an extreme environment, and to shield the fiber exterior against mechanical abrasion^5,6^. Plant fibers have better specific properties, i.e. the ratio of mechanical properties per mass, which makes them good candidates for lighter materials. As renewable and recyclable materials, they are environmentally friendly^7^. Suddell and Evans^8^, reported on the categories of natural fibers with their unique properties for consideration in the development of fiber based composites. Few of the natural fibers include coir, cotton, wood, ramie, coconut leaves, jute, sisal, hemp etc. Their choice as candidate reinforcement material is informed by the potential area of application among other criteria. Natural fiber such as hemp, kenaf and flax have been successfully utilized in the design of polymer based composites. This is due to their excellent functional properties with their positive economic implication^8^.

Polypropylene (PP) is a known thermoplastic “addition polymer” derived from the combination of propylene monomers. It is reported to be one of the most widely used plastics in the world and is ranked third in usage after polythene^9^. PP finds its application in many house wares, packaging for user products, appliances, bottles, special devices like living hinges, automotive industry, textiles and heater ducts where high heat deflection is required^9^. Essential properties possessed by PP include good transmissivity, elasticity, toughness property, fatigue resistance and good chemical resistance^10^.

Dombeya buettneri fiber (DBF) is a readily available plant fiber grown in Madagascar and some other African countries like Nigeria. The stem releases a very sticky fluid when cut which exude gums and resins. The stem bark is very fibrous from where fiber can be extracted manually to make rope, nets and fishing apparatus^11,12^. Detailed information on the fiber properties is lacking as it has only been used extensively in the field of medical sciences but not for materials development or engineering application as in the case of this present work. In traditional medicine, it finds a unique impart, the leaf has been used in the treatment of gastrointestinal problems confirmed in experiments with rats^12^. In DR Congo for instance, they are made as cords to support basket on the back for carrying loads. Hence, the choice of DBF as lignocellulosic reinforcement in polymer composites becomes more appealing due to their inherent strength^12^.

Graphite is one of the softest metals that is carbon in its stable form. It has good thermal and electrical conductivity and makes a good lubricant. Graphite powder is soft but does not have elastic or stretching properties. Graphite exhibiting an anisotropic nature within the in-plane and out-of-plane of its atoms with higher elastic modulus parallel to the plane as well as compared to the perpendicular^13^. It is an allotrope of carbon with high thermal conductivity property. Literature has it that at ambient temperature and pressure, carbon often exhibits a lowest energy state^14^. However, at higher temperature (~ 2,500 °C) carbon and graphite display a good strength and stiffness properties. Composites developed from carbon fiber are mostly used in space, aeronautical, bio-medical, and industrial application. Currently, they are mostly used in commercial and defense areas^15^ with the availability of inorganic fillers. Previous works from researchers have established the use of hybrid reinforcements for composite development^16–19^, however, dombeya buettneri fiber and graphite has not been used. The current work examines the influence of dombeya buettneri fiber and graphite particles on polypropylene. Part of the novelty of the research was the synergy between newly applied vegetable fiber and an established synthetic particle. This was done to extend the frontiers of knowledge in the use of natural fiber and the areas of applications^20–23^. These reinforcements were selected so as to explore their effect on polypropylene matrix, which is widely used among the thermoplastic material.

## Experimental

### Materials

**Plate 1:**
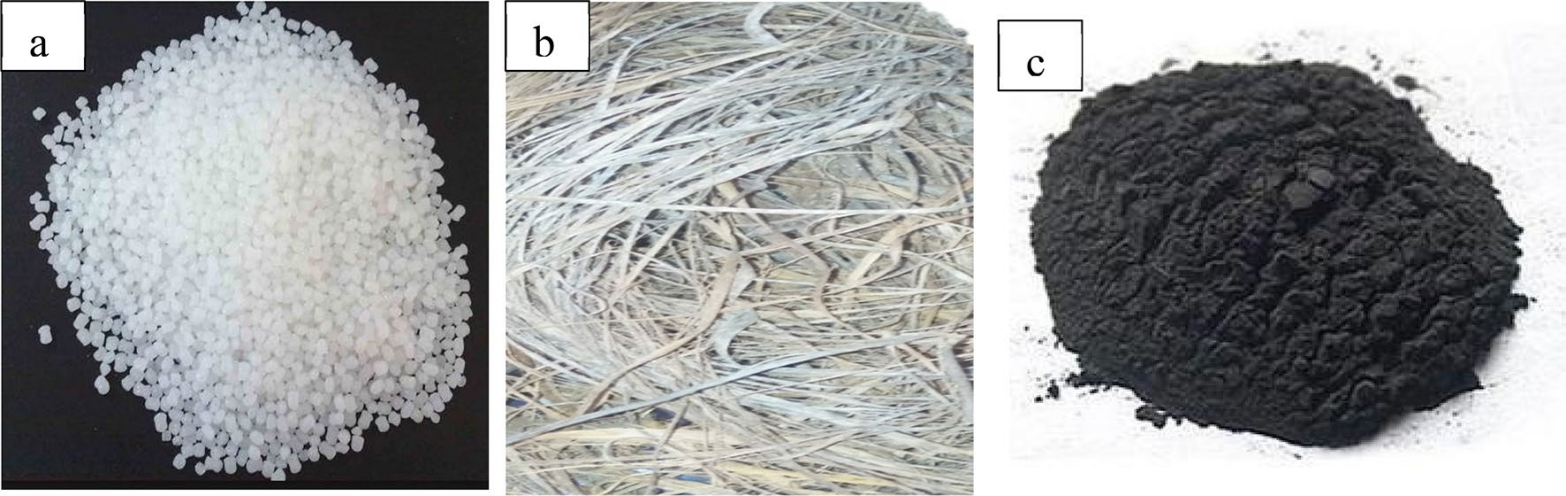
(**a**) Polypropylene pellet; (**b**) Dombeya Buettneri fiber; (**c**) graphite particle.

The chemicals; dioctyl phthalate (DOP), zinc stearate and sodium hydroxide were procured from allied stores in Edo State, Nigeria while the homopolymer polypropylene (PP) shown in Plate 1(a) from REPOL, a Reliance Polymer Product Manufacturer in India, was obtained from chemical and allied store in Lagos State, Nigeria. The dombeya buettneri fiber shown in Plate 1(b) was acquired from a farm plantation in Akure, Ondo State, Nigeria. The graphite powder shown in Plate 1(c) which is flake-like and very fine with an average particle size of ~ 10 μm was procured from Pascal Scientific in Akure, Ondo State, Nigeria.

### Procurement and preparation of dombeya buettneri fiber

One of the prime materials used for this research was dombeya buettneri fiber. The fiber was extracted by removing the bark from the stem, followed by manually separation of the strands and sun dried for 5 days. The fiber was treated by immersion in a solution of 1 M NaOH at 50 °C for 4 h in a shaker water bath. The treated fiber was washed with tap water and rinse with distilled water till neutral status was ensured after confirmation by litmus paper. The treated fiber was sun dried for 5 days during the dry season until the moisture content is removed.

### Development of the composite samples

The composite was developed using compression molding process by randomly dispersing the dombeya buettneri fiber and graphite particle in the polypropylene matrix in predetermined proportions. The working condition for the production of the hybrid composite was 170 °C for 7 min at 5 bar. Compounding of samples was carried out to ensure an even distribution of the reinforcement in the matrix and a proper blend, followed by reproduction of the sample but with the addition of plasticizer and stabilizer using the same working parameters. The ratio of dioctyl phthalate to zinc stearate that was used as plasticizer and stabilizer respectively was 2:1. The fiber after compounding was cut to short fiber lengths of average size 6–8 mm during grinding. The composite composition was presented in Table 1.

**Table 1:** Formulation of the composite.

Sample denotation	PP (wt%)	PP (g)	DBF (wt%)	DBF (g)	GP (wt%)	GP (g)
Control	100	173	–	–	–	–
A	80	138.4	18	31.14	2	3.46
B	80	138.4	15	25.95	5	8.65
C	80	138.4	12	20.76	8	13.84
D	80	138.4	9	15.57	11	19.03
E	80	138.4	6	10.38	14	24.22
F	80	138.4	3	5.19	17	29.41
GP	80	138.4	–	–	20	34.6
DB	80	138.4	20	34.6	–	–

Total weight of the materials that filled the mould was 173 g.

### Property test

#### Tensile test

The tensile tests were conducted according to ASTM D3039/D3039M-17^24^ standard on a universal testing machine Instron series 3,369 model. The specimens with dimensions 90 × 10 × 5 mm dumb-bell shape were used. The test was conducted at a crosshead speed of 5 mm/min using 10 kg load cell. To ensure precision and reliability of tensile test results, three repeatability tests were performed for each of the developed samples.

#### Flexural test

Three points bending test was used to evaluate the flexural property of the samples. Universal testing machine series 3,369 model was used to carry out the flexural test in accordance to ASTM D790-03^25^ standard. A sample cut into 150 × 50 × 5 mm was placed in grip of the machine and was stretch at a test speed of 5 mm/min over a span of 65.00 mm. Three samples were tested for each composition and the average value was used as the representative values.

#### Impact test

The notched Izod impact test was conducted in accordance with ASTM D 256-10^26^ a standardized test method for determining the Izod Pendulum impact resistance of plastics. The test was carried out using a Hounsfield balanced impact testing machine, serial number 3915, model number h10-3. Impact test dimension of 64 × 11 × 3 mm was notched at the center. Samples were placed horizontally on the machine, maintaining a distance of 60 mm between lines of supports. The test samples were placed in a cantilever position, clamped upright with a V- notch at the level of the top of the clamp. The machine pendulum hit the test piece and was allowed to fall freely to a fixed height.

#### Hardness test

Hardness test was conducted on the specimen using a Shore D hardness tester in accordance with ASTM D2240-00^27^. The samples were placed on the flat surface of the tester stand and indented. Four values were obtained by indenting the samples in four different places and the average value was used for analysis.

#### Abrasion test

Wear test was performed to evaluate the wear property of a material to determine the suitability of the composite for specific wear application. The samples were evaluated using the Taber abrasive tester model TSC-A016 in accordance with ASTM D 1044-13^28^, a standardized test method for resistance of transparent plastics to surface abrasion. Prior to the test, the initial weight of the sample was measured. Each sample was mounted on the Taber abrasive tester operated at 150 rpm for 10 min, and then the weight post abrasion testing was taken. A mould of 100 mm diameter × 4 mm thickness was used to produce the wear samples. The wear index was determined from Eq. (1).

1 $$\mathrm{W}\mathrm{e}\mathrm{a}\mathrm{r} \mathrm{I}\mathrm{n}\mathrm{d}\mathrm{e}\mathrm{x}, \left(\mathrm{W}.\mathrm{I}\right)= \frac{\mathrm{i}\mathrm{n}\mathrm{i}\mathrm{t}\mathrm{i}\mathrm{a}\mathrm{l} \;\;\mathrm{w}\mathrm{e}\mathrm{i}\mathrm{g}\mathrm{h}\mathrm{t} - \mathrm{f}\mathrm{i}\mathrm{n}\mathrm{a}\mathrm{l} \;\;\mathrm{w}\mathrm{e}\mathrm{i}\mathrm{g}\mathrm{h}\mathrm{t} }{\mathrm{t}\mathrm{i}\mathrm{m}\mathrm{e}\; \mathrm{o}\mathrm{f}\; \mathrm{t}\mathrm{e}\mathrm{s}\mathrm{t}\; \mathrm{c}\mathrm{y}\mathrm{c}\mathrm{l}\mathrm{e} } \times 100$$

The wear index indicates the rate of wear calculated by measuring the loss in weight in milligrams per test cycles of abrasion.

#### Thermal conductivity test

Thermal test was carried out using the Lee’s disk apparatus to determine the thermal conductivity of the developed composite in accordance with ASTM E1530-19^29^. The thermal conductivity analysis was carried out at a temperature range of 50–80 °C and at such no temperature or thermal degradation was noticeable as the material developed was not tested at a temperature close to the activation of degradation^30^. To determine the thermal conductivity of the samples, Eq. 2 was used.

2 $$k=\frac{m cp \left({\varnothing }_{1}- {\varnothing }_{2}\right)4x}{\pi {D}^{2}\left({T}_{1}-{T}_{2}\right)t}$$

where, K is the thermal conductivity; m is the mass of the disk, 0.0078 kg; cp is the specific heat capacity of the disk, 0.91 kJ/kg K (910 J/kg K); ∅1, ∅2 is the initial and final temperature of disk B; D is the diameter of the sample, 0.04 m; x is the thickness of the sample, 0.003 m; T1, T2 is the temperature of disk A and B in Kelvin Temperature of disk A and B in Kelvin; t is the final time taken to reach a steady temperature.

#### Water absorption test

Water absorption tests were carried out in accordance with ASTM D5229M-12^31^. To carry out the test, 250 cm^3^ of water media was poured into clean plastic containers. The initial weight of each of the sample was taken using chemical weighing and readings were taken every day for seven days. To take the readings, the samples were brought out, cleaned with clean cloth before been weighed. The data collected was used to determine the weight gained using the formula in Eq. (3).

3 $$W(g)={W}_{t-}{W}_{0}$$

where *W* (*g*) is weight gain per hour, ${W}_{0}$ and ${W}_{t}$ are the oven dry weight, and the weight of the sample after time t, respectively.

## Results and discussion

### Mechanical properties

**Figure 1:**
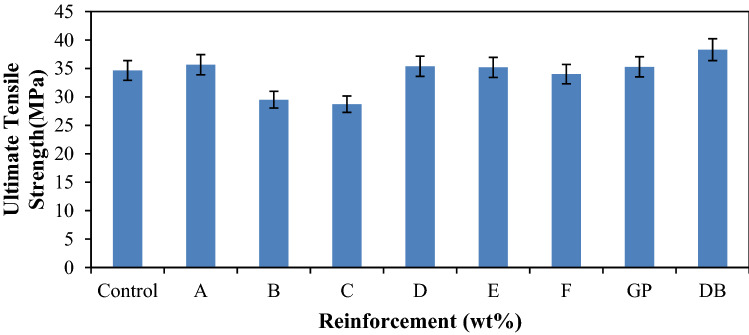
Variation of ultimate tensile strength for control, single and hybrid reinforced polypropylene composites.

Figure 1 shows the ultimate tensile strength (UTS) responses of the developed composite and the control samples where it was discovered that the UTS tends to reduce from A–F. Samples A–F were the hybrid composite samples and, sample A being the blend of 18 wt % of DBF and 2 wt % of GP, having value of 35.66 MPa as compare to the control that has a value of 34.64 MPa was the best. The difference amounted to 3% enhancement in property. However, higher strength was achieved with single reinforcement content of DBF with a value of 38.30 MPa that culminated to 11% enhancement. The improved UTS observed from DBF reinforced sample may be due to the alkaline treatment that was carried out on the fiber as stated in previous findings that such treatment usually enhances the tensile properties of natural fiber^23^. In the hybrid composites, the UTS tend to decrease as the DBF reduces from sample A to F. The observed trend can also occur due to improper transfer of load from the matrix to the reinforcement resulting from improper interfacial adhesion at the reinforcement/matrix phase or when the ratio of the two constituents are inappropriate; consequently, weak interface will occur. In this hybrid system, the interfacial phase seems more complex due to the origin and shape of the reinforcements which are synthetic/natural and particulate/fiber, respectively. From the results obtained for single and hybrid reinforced composites, it shows that, the presence of graphene particulate in high proportion tends to decrease the UTS compared to dombeya buettneri fiber. This may be due to inadequate wettability of the particles owing to higher filler loading that resulted in poor fiber-particle–matrix adhesion and, thus, decreased tensile strength. The decrease in the tensile strength as the graphite particles increased beyond hybrid composite sample A can be due to the interference of DBF and GP in the mobility or deformability of the matrix. While the improved tensile property of the hybrid reinforced composite may be attributed to the fair distribution of the DB fiber in the polypropylene matrix resulting in strong DBF-GP-PP matrix interaction. Similar results have been reported by previous researchers^32–34^.

**Figure 2:**
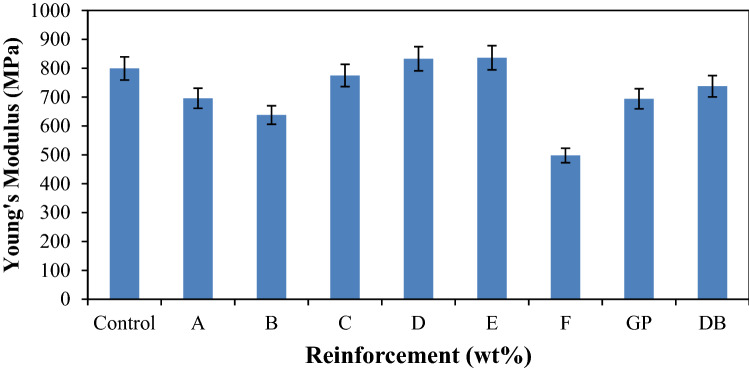
Variation of Young’s modulus for control, single and hybrid reinforced polypropylene composites.

Figure 2 shows the response of the developed composites and the control sample to Young’s modulus. The Young’s modulus of the composites tends to increase with increase in the graphite content up till a maximum value (6 wt% DBF: 14 wt% GP) for hybrid composite sample E that has the best Young’s modulus (836.15 MPa). This response revealed that GP enhances the stiffness of the developed composites compared to the DBF. Particulate reinforced polymer matrix composites have been reported to possess improved tensile modulus in previous research. Oladele et al.^35^ reported from their results that, the enhancement of tensile modulus is not dependent on the particle size but on particle loading. Also, stated was that the occurrence of improved stiffness with corresponding increase in filler loading in particulate-filled polymer composites has been attributed to the fact that most inorganic fillers exhibit rigidity that is significantly higher than that of the polymer matrix. These observations are in agreement with the investigation of Fu et al.^36^ where they studied the effect of particle size on the tensile modulus of particulate–polymer composites^37^. Therefore, as the filler loading increases, the degree of obstruction increases, this in turn increased the stiffness. This is also in agreement with the findings of Fardausy et al.^38^ that the tensile modulus of the fabricated composites increases with increase of filler addition. The results obtained in this study attributed that the fillers had created some reinforcing effect and had been responsible for the increase in Young’s modulus^34^. It can be deduced that the samples that has high modulus of elasticity has better stiffness property compared to others and this is due to the presence of the higher proportion of graphite particles^13^. In this research, about 5% enhancement was achieved.

**Figure 3:**
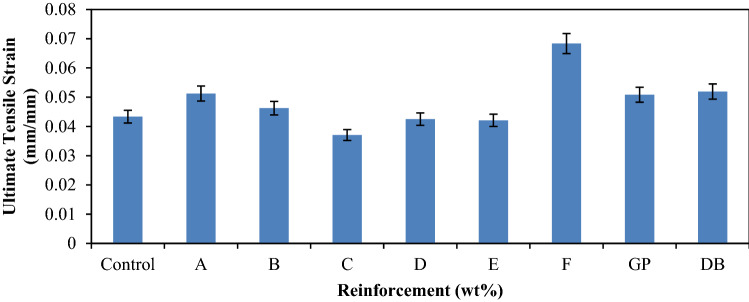
Variation of tensile strain for control, single and hybrid reinforced polypropylene composites.

Figure 3 shows the tensile strain of composites with various reinforcements and that of control. It was observed that sample F has the highest strain value of 0.068 mm/mm. Hybrid composite sample F which is the blend of 3 wt% of DBF and 17 wt% of GP exhibits the maximum strain compared to control which culminated to about 57% enhancement in property. From the graph it was observed that the addition of GP and DB fiber as a single reinforcement in the matrix has almost the same effect on the strain of the composite with values; 0.051 and 0.052 mm/mm, respectively. However, as the DBF content decreases from A – D, there is a progressive decrease in the tensile stain. Conversely, at a particular mix ratio of DBF and GP content (3:17), in which GP becomes more than that of the DBF, the tensile stain increases rapidly. The observed performance was due to the properties of the graphite^13,14^. This implies that the composition of sample F gave the best propensity for high strain before failure.

**Figure 4:**
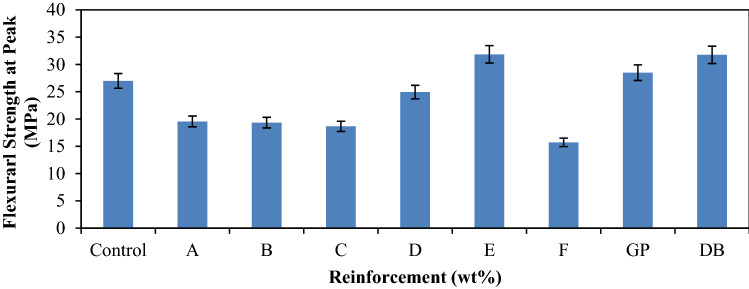
Variation of flexural strength at peak for control, single and hybrid reinforced polypropylene composites.

Figure 4 present the findings based on the influence of bending load on the samples. It was discovered that most of the developed hybrid composites have low flexural strength compared to the single reinforced composites and the control sample. However, sample E (6 wt% DBF:14 wt% GP) from among the hybrid composites has the highest value (31.84 MPa) which culminated to about 18% enhancement. The same sample was the one with the best Young’s modulus as shown in Fig. 2 where it was deduced that the sample gave the best composition for high resistance to early deformation. High stiffness of this material was responsible for its good flexural strength. Considering the flexural strength of single reinforced composites, it was observed that both aided the enhancement of the property. However, better enhancement was achieved with the addition of dombeya buettneri fiber with a value of about 31.74 MPa. Actually from the results, much improvement was not achieved from the hybrid composite E with respect to this single reinforced composites. Therefore, it will be more economical to use the single reinforced composite composition if bending strength is the major challenge to be encountered in service.

**Figure 5:**
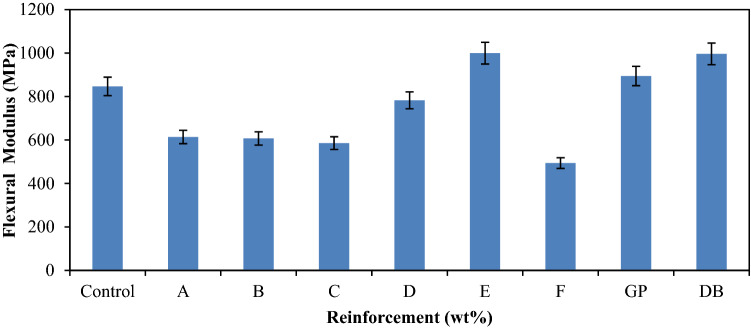
Variation of flexural modulus for control, single and hybrid reinforced polypropylene composites.

Figure 5 shows the variation of flexural modulus of the control, single and hybrid reinforced polypropylene composites in which similar trend to Fig. 4 were noticed. Sample E marginally gave the best flexural modulus value of 999.55 MPa compared to single reinforced composite from It DB fiber which a value of 996.41 MPa. Addition of GP and DB fiber as a single reinforcement gave improved modulus, respectively to show that sample with single reinforcement compete favorably with hybrid composite sample E with the blend of 6 wt% DBF and 14 wt% GP. Nevertheless, the influenced of the reinforcements on the PP with a flexural modulus value of 846.67 MPa amounted to 18% enhancement. The improvement in flexural modulus was attributed to the good stiffness that was induced into the matrix by the reinforcements.

**Figure 6:**
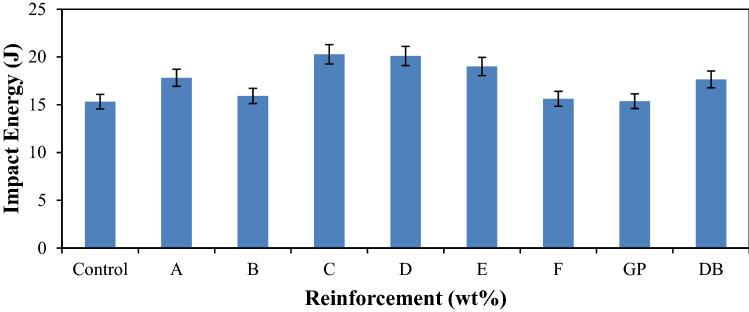
Variation of impact energy for control, single and hybrid reinforced polypropylene composites.

The effect of different proportion of reinforcement content on the impact energy of the developed composites and the control sample was shown in Fig. 6. It was observed from the results that all the developed composites have improved impact energy than the control sample. The impact energy of the hybrid composite samples was increasing as the GP content was increasing up to a certain proportion before decreasing. This trends were represented by samples A–C and D–F. The gradual decrease in impact energy after hybrid composite sample C was due to the increase of GP contents that might have led to partial agglomeration of the GP in the matrix. It was observed that the blend of 12–9 wt% of DB fiber and 8–11 wt% of GP gave the optimum results with sample C emerging as the best. This behavior indicates improved fiber-particle–matrix adhesion due to proper transferring of loads from the matrix to the reinforcements. This is in tandem with the findings of Shrivastava and Agrawal^39^ that fibers play an important role in the impact strength; they resist the crack propagation and act as a load transfer medium. Similar findings were reported by other researchers^37,40^. The maximum impact energy obtained in this research was 20.28 J with hybrid composite sample C which connotes about 32% enhancement in this property. Considering the impact property of the single reinforced composites, it was observed that the addition of GP in the matrix led to improved impact energy of the composites. However, the improvement was more appreciable with the addition of DB fiber.

**Figure 7:**
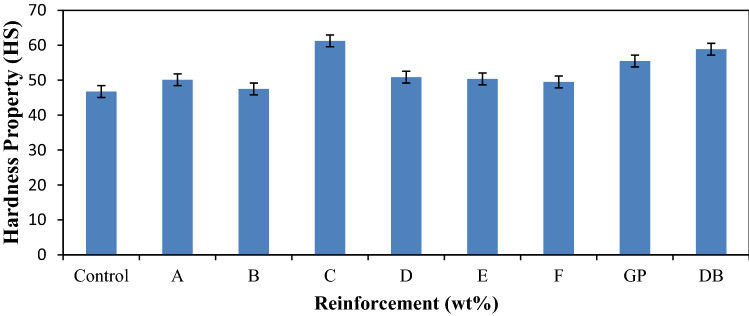
Variation of hardness property for control, single and hybrid reinforced polypropylene composites.

Surface hardness of the composites is one of the most important factors that directly have effect on the wear resistance of the composites. From Fig. 7, it was clear that the composites samples have improved resistance to surface indentation (hardness) property as compared to control. The single reinforced DB fiber composite increased the hardness than that of graphite with values 58.88 HS and 55.50 HS, respectively. The improvement with single reinforced DB fiber composite compared to the control was about 26% enhancement. However, the synergetic effects of the reinforcements were responsible for the enhancement in sample C with high DB fiber content than graphite particle (12:8). This development could be linked to the better adhesion of the matrix to the fiber due to surface modification by NaOH treatment. The hardness property reaches its maximum value of 61.25 HS at hybrid composite sample C; this was 31% enhancement in the hardness of the material. This was attributed to the fact that hardness is a function of the relative fiber content. Increases in the weight percentage of the DB fiber and stiffness of GP within matrix of hybrid composite sample C is almost at equilibrium to enhance distribution of stress which offers resistance to indentation. The hardness properties of a composite were enhanced as a result of the decrease of flexibility and increase of stiffness of a composite^41^.

### Wear property

**Figure 8:**
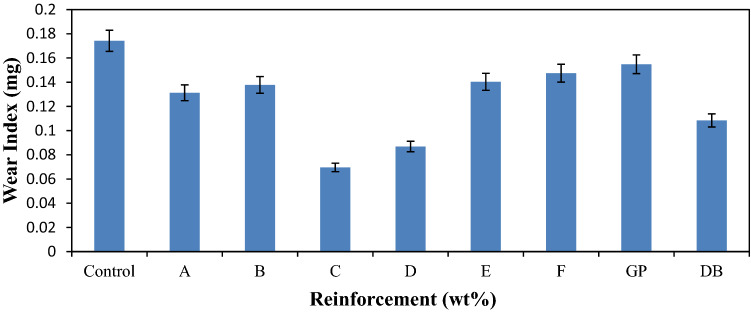
Variation of wear index for control, single and hybrid reinforced polypropylene composites.

Figure 8 revealed the response of the materials to wear condition. It was observed from the results that, samples with higher wear index values has high wear rate and low wear resistance as depicted by the control sample that has the highest value of about 0.174 mg. There was improvement in the wear property of the composite due to the addition of reinforcements in the development of single and hybrid reinforced composites, respectively. It was discovered that composite samples with high DBF content have good wear resistance properties. The hybrid composite samples with an optimum blend of 12–9 wt% DBF and 8–11 wt% GP which are samples C and D are of good results. However, sample C has the best wear resistance with wear index of 0.069 mg. The chemical treatment of the DB fiber enhance proper bonding between the matrix and the fiber at their interface and, hence, improve the wear property of the single dombeya fiber reinforced composite and that of the hybrid composite samples. From the analysis, the observed enhancement in the wear property from the best hybrid composite sample was about 150% than that of the control.

### Thermal conductivity property

**Figure 9:**
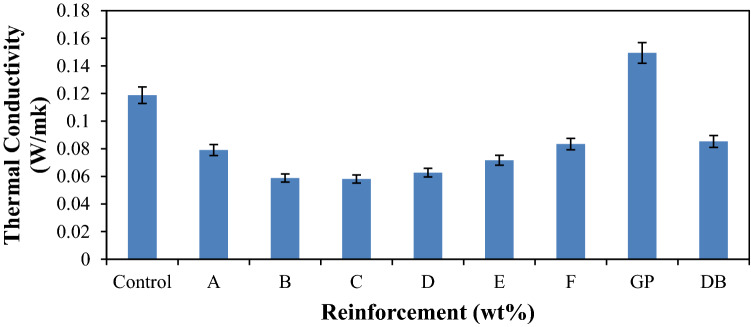
Variation of thermal conductivity for control, single and hybrid reinforced polypropylene composites.

Thermal conductivity is a measure of the rate of heat flow through one-unit thickness of a material subject to a temperature gradient^42^. A material with higher thermal conductivity implies that it is more conductive and lesser value implies a good insulating property of the material. Thermally conductive polymer composites are typically developed by introducing fillers of high thermal conductivity, such as lightweight carbonic materials^43–45^. It was observed in Fig. 9 that there was a decrease in the thermal conductivity value of the entire hybrid composite formed as compared to the control sample. There was an initial decrease in the thermal conductivity of the hybrid composites from sample A–C followed by an increase from samples D–F. This increase is attributed to the presence of graphite in the blend, as the GP content increases beyond sample C, there is a corresponding increase in the thermal conductivity value. This follows the finding of Mokhena et al.^46^, that polymer/graphite composites exhibited a high thermal conductivity and an electrical conductivity at a fairly low concentration, amongst other fillers, graphite features unique properties such as a high thermal and electrical conductivity, a low coefficient of thermal expansion, a high thermal shock resistance, improved stiffness and an increased strength. From the results, it was noticed that the thermal conductivity varies with the reinforcement content. It was discovered from the mechanical properties results that the reinforcement materials toughens the matrix and, hence, prevent the composites from conducting the thermal energy as more barriers are developed in transporting from one side of the sample to the other^47^. The moderate thermal conductivity value of the hybrid composites was a proved that there is a synergetic effect of GP and DB fiber on the compositions. When the single reinforced composites are compared, the DB fiber reinforced composite and GP reinforced composite has values of 0.085 W/mk and 0.149 W/mk, respectively. This signifies that DB fiber is more insulating than the GP. From the result, it was observed that the blend of 12 wt% of DB fiber and 8 wt% of GP gave the optimum result of 0.058 W/mk which amount to about 105% enhancement from sample C as the best insulating materials. Conversely, GP reinforced composite was the best in terms of conductivity with about 25% improvement when compare with the control sample (0.119 W/mk).

### Water uptake

**Figure 10:**
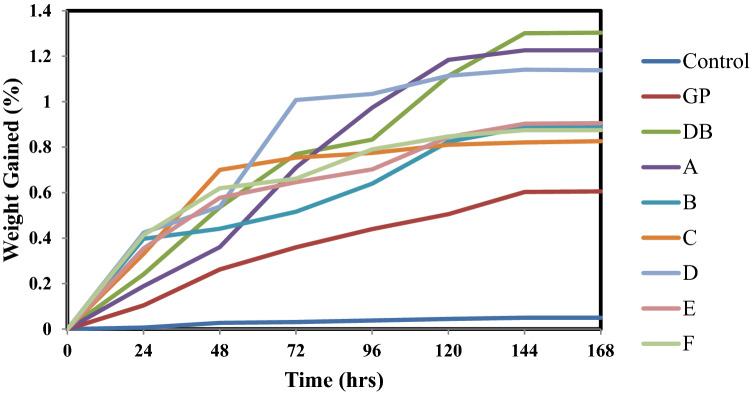
Variation of percentage weight gain per hour for Control, Single and Hybrid Reinforced Polypropylene Composites.

Figure 10 shows the trend of moisture up-take per hour for control, single and hybrid reinforced polypropylene composite samples. From the graph it can be seen that all the composites absorb water rapidly and linearly at the initial stage before the saturation level was attained at 144 h where further increase in water absorption was not noticeable.

According to Fick’s law (J) which measures the amount of substance that will flow through a unit area during a unit time interval, diffusion of water in polymer also occurred in such mode. Water is a transmission medium for oxygen and ions, hence, diffusion of water in polymers is an important mechanism to be understood^48,49^. The first thing to do in order to understanding water diffusion is to examine diffusion path and relaxation conditions as given by Fick’s law^49^. Though, this is challenging because of the complexity of polymer structure and compositions, and the difficulty in evaluating the details of molecular-level for such a small sample^50^. The second assumption of ideal Fick’s diffusion is that the polymer variation caused by water diffusion can be negligible, which is absolutely not acceptable for most experiments. According to the relative rate of polymer relaxation compared to that of water diffusion, three types of water diffusion are possible as stated by Frisch^51^. These three types were seen in the results presented in Fig. 10.

The diffusion rate from 0 to 48 h agreed with Case I (Fick’s diffusion, n = 0.5) where polymer relaxation is much faster than water diffusion, and the diffusion is followed by an instantaneous response of the system, resulting in Fick’s behavior. The instantaneous response of the system requires large flexibility of the polymer chains in the system, that is, the polymer is in a rubbery state. In this case, diffusion is controlled by the diffusion coefficient^49^.

The diffusion rate from 48 to 144 h agreed with both Case II (n = 1) and Super Case II (Irregular diffusion, n > 1). Under these conditions, the rate of diffusion is much faster than that of relaxation initially which marks the innermost limit of water diffusion, and it is the boundary between the (stressed equilibrium) swollen gel and the glassy core of polymers. At this moment, the swelling of polymers occurs. This was followed by rate of diffusion that is equal to that of relaxation (Irregular diffusion, n > 1) where evolution, under certain circumstances, of Super Case II transforming from Case II makes it somewhat uncertain^49,52,53^.

The final stage from 144 to 168 h in these results revealed that, the diffusion or absorption of water become constant or fixed and it is referred to as saturation point.

The total moisture uptake of the control sample after 168 h was 0.05% while the sample with least moisture absorption among the composites was from single reinforced composite GP was with a value of about 0.61%. DBF reinforced composites was the sample with the highest moisture absorption of about 1.30%. This was followed by sample A being the blend of 18 wt% of DBF and 2 wt% of GP with a value of about 1.23%. These results showed that the DB fiber has high affinity for water than GP as confirmed in hybrid composite sample F (a blend 3 wt% DB fiber and 17 wt% GP) having total moisture uptake of 0.87%. For the hybrid composites, the water absorption tends to decrease with increase in the GP content with the exception of hybrid composite sample D. Hybrid composite sample C (a blend 12 wt% DB fiber and 8 wt% GP) has the lowest water up take amidst the hybrid composite developed which is 0.83%. This composition and the feat can be due to the interplay of DB fiber and GP being at equilibrium to effectively reinforce the polypropylene matrix. Considering the single reinforced composite, GP has a higher resistance to moisture absorption than DB fiber. Thus, it was noticed that GP was hydrophobic while the DBF was hydrophilic in nature. However, the synergetic influence of these reinforcements aids a better material for structural applications with respect to water intake.

### Microstructural analysis

**Plate 2:**
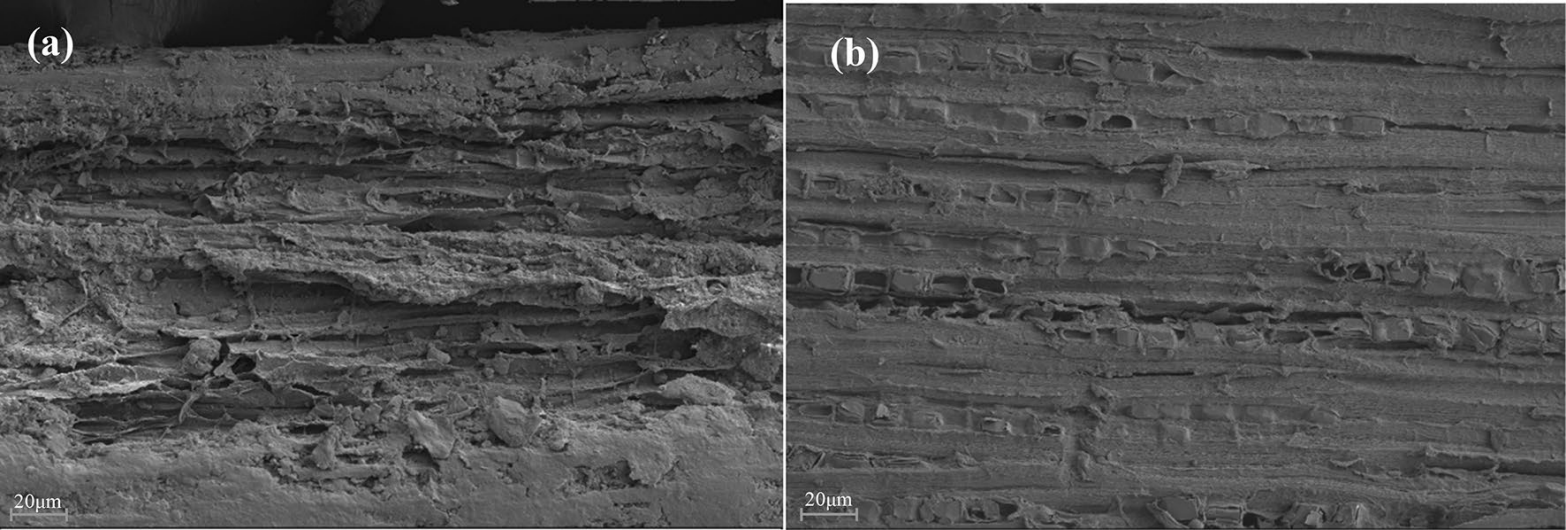
SEM image of the untreated and treated *Dombeya Buettneri* fibers; (**a**) untreated fiber and (**b**) 1 M NaOH treated fiber.

The surface morphology of the fibers as observed from the SEM in Plate 2 revealed that the untreated DB fiber surface was rough and has many cavities which look like an eroded surface. This may be due to the extraction process which might have left a lot of unwanted constituents on the surface of the fiber. A common feature in the morphology the fiber is that in both cases (Plate 2a) and (Plate 2b) voids could be observed, but well reduced to a considerable level in the treated sample. This supports the idea that natural fibers might act as a potential nucleation site for bubbles^54^. The chemical treatments reduce the level of surface roughness compared to the untreated DB fiber. Alkali treatment has been discovered to be responsible for the removal of some hemicelluloses, lignin, glue and other extractives in fiber bundles which yields higher percentage of α-(alpha) and cellulose in natural fibers^23^.

**Plate 3:**
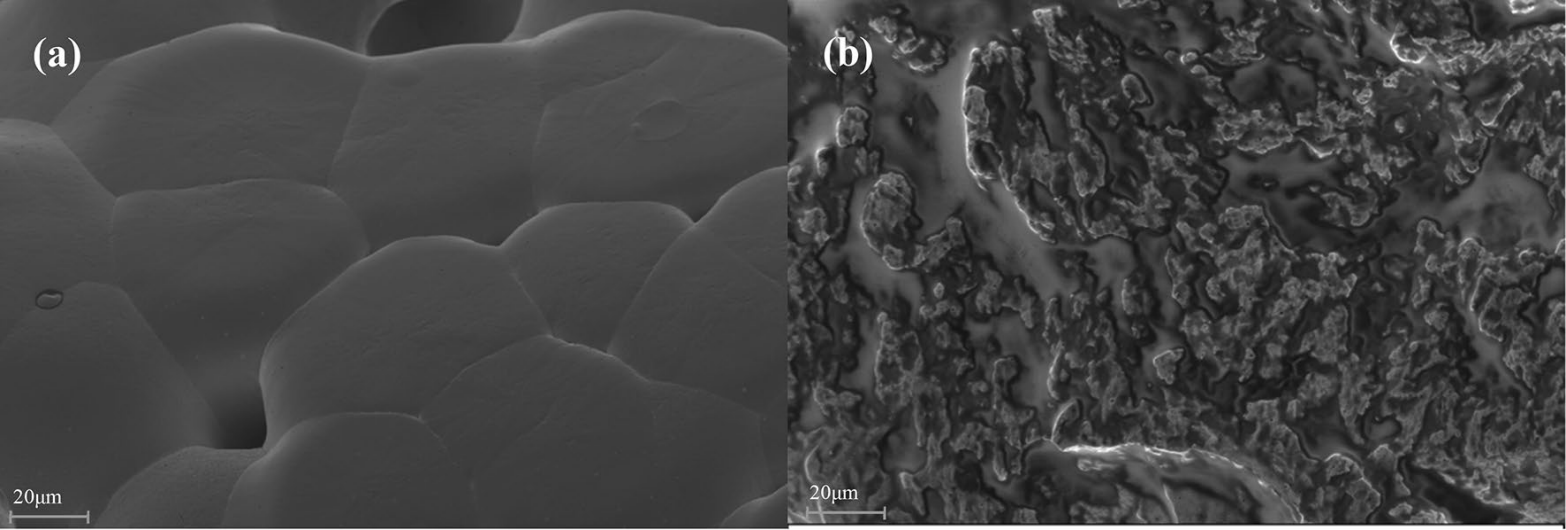
SEM image of the fractured surface of the composite; (**a**) PP/*Dombeya* and (**b**) PP/graphite.

**Plate 4:**
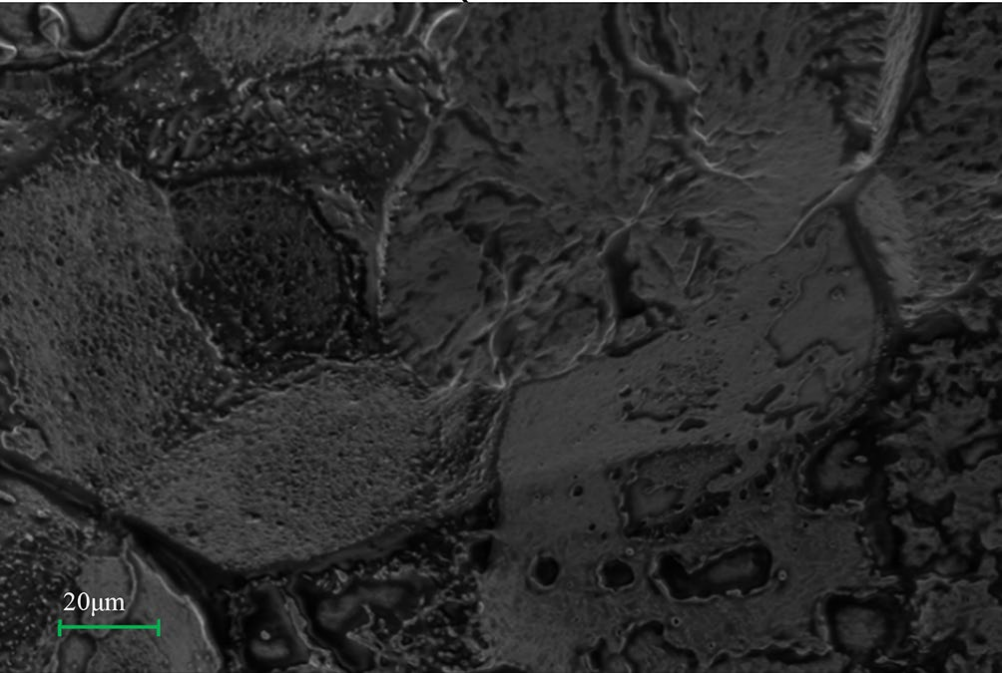
SEM image of the fractured surface of the hybrid composite.

Plate 3(a) and (b) show the surface morphology of PP/DB fiber and PP/GP, respectively. It can be observed that there is a significant difference in the surface morphology of the composites. The PP/DB fiber was observed to have no evidence of fiber pull out at the fractured surface morphology but well imbedded within the PP matrix while the PP/GP composite fractured surface revealed a well dispersed graphite particle within the PP matrix as the dark phase of the image. Different features can be observed from the SEM image of the hybrid composite in Plate 4. Part of the reasons for the observed features was the influence of chemical treatment on the surface condition of the fiber and well dispersed particles within the PP matrix. The synergetic effect that yielded improved properties in the developed composites was observed in the image as the individual features in 3 (a) and (b) were also noticed in Plate 4 image. The proper bonding at the interface of the developed hybrid composite due to appropriate wetting and distribution gave the hybrid composite superior properties than the single reinforced composite and the control sample.

## Conclusions

Chemical treatment was noticed to reduce the roughness of the untreated DB fiber surface, thereby, making the surface more suitable for good fiber-matrix interaction due to de-lignification of the surface. The hybrid composites emerged as the best material in most of the properties examined except ultimate tensile strength, water absorption and thermal conductivity compared to other samples. Sample denoted as C which is the blend of 12 wt% DB fiber and 8 wt% GP has the optimum properties from all the samples being 31%, 32%, 105% and 150% enhancement in hardness, impact, thermal and wear properties, respectively followed by sample denoted as E which is the blend of 3 wt% DB fiber and 17 wt% GP with best results from flexural properties and Young’s modulus. With the good wear resistance and thermal insulating properties of the hybrid composite samples, it can find application in automobile industries for interior application like door panel and can also be used as protection for pipelines vessels. Diffusion of water into the composites also obeys Fick’s law where sample C was seen to be the best among the composites.

## Data Availability

The data that support the findings of this study are available within the manuscript.
